# Influence of anthropometric features on peroneus longus graft diameter in Anterior Cruciate Ligament reconstruction: A cohort study

**DOI:** 10.1016/j.amsu.2019.10.023

**Published:** 2019-11-01

**Authors:** Sholahuddin Rhatomy, Henry Tanzil, Riky Setyawan, Camilla Amanda, Krisna Yuarno Phatama, Jeffrey Andrianus, Tedjo Rukmoyo, Bambang Kisworo

**Affiliations:** a Department of Orthopaedics and Traumatology, Dr. Soeradji Tirtonegoro General Hospital, Klaten, Indonesia; b Soeradji Tirtonegoro Sport Center and Research Unit, Dr. Soeradji Tirtonegoro General Hospital, Klaten, Indonesia; c Faculty of Medicine, Public Health, and Nursing, Universitas Gadjah Mada, Yogyakarta, Indonesia; d Department of Orthopaedics and Traumatology, Dr. Saiful Anwar General Hospital, Malang, Indonesia; e Faculty of Medicine, Universitas Brawijaya, Malang, Indonesia; f Department of Orthopaedics and Traumatology, Dr. Soetomo General Hospital, Surabaya, Indonesia; g Faculty of Medicine, Universitas Airlangga, Surabaya, Indonesia; h Department of Orthopaedics and Traumatology, Dr. Sardjito General Hospital, Surabaya, Indonesia; i Department of Orthopaedics and Traumatology, Panti Rapih Hospital, Yogyakarta, Indonesia

**Keywords:** ACL, Graft diameter, Peroneus longus, Patient characteristics

## Abstract

**Background:**

Anterior Cruciate Ligament (ACL) is the most common ligament injury during sports activities that was treated with ACL reconstruction. Nowadays, peroneus longus is used in ACL reconstruction. However, it is difficult to predict the peroneus longus graft diameter for ACL reconstruction. Thus, preoperative measurements are very important to predict peroneus longus autograft for ACL reconstruction.

**Methods:**

A cohort retrospective study was conducted using consecutive sampling method from February 2016 until October 2017 in our center. We recorded patients’ characteristics include gender, age, body weight, height, and Body Mass Index (BMI) preoperatively. We measured peroneus longus graft diameter intraoperatively, and analysed data using Spearman correlation.

**Results:**

Thirty-nine patients met inclusion criteria. There were 28 males and 11 females in the peroneus group. From the patients' mean characteristics, age was 25.10 ± 9.16, body weight 71.23 ± 14.17, height 169.13 ± 8.81, and BMI 20.96 ± 3.44. Intraoperative peroneus longus diameter measurement was 8.56 ± 0.82. Spearman correlation showed significant correlation between intraoperative peroneus longus diameter with patient's height, body weight, and BMI with *p* < 0.05.

**Conclusion:**

Patients’ characteristics including gender, height, weight, and BMI in preoperative measurements can predict peoneus longus graft diameter intraoperatively.

## Highlights

• Gender.
• Height.
• Weight.
• Body Mass Index.
• Peroneus longus tendon diameter.
• Patients' characteristics in preoperative measurements can predict graft diameter intraoperatively.

## 1 Introduction

Anterior Cruciate Ligament (ACL) is one of many ligaments in the knee that is commonly injured in sports and accidents [1]. Incidence of ruptured ACL is higher in females than in males [2]. Recently, ACL reconstruction has become a common procedure to treat torn ACL in highly active person [2]. ACL reconstruction goals are to restore knee stability, relieve symptoms, particularly pain and instability, and return the patients to their daily activity as before injury [1].

Graft that most common used in ACL reconstruction is hamstring tendon and Bone-Patellar Tendon Bone (BPTB). In previous study, there are many ways to predict graft size diameter of the hamstring tendon such as Magnetic Resonance Imaging (MRI). MRI scanning can be used to determine the diameter of the hamstring graft that will be used for ACL reconstruction. With a cross-sectional area >22 mm^2^, a diameter of hamstring > 8 mm can be obtained [4]. The other way was used anthropometric examinations such as height and weight is relatively easier and still relevant to determine the diameter of the hamstring [5].

Recent studies reported that the peroneus longus tendon autograft usage for knee ligament reconstruction like ACL [6,7], Posterior Cruciate Ligament (PCL) [8]. This study aimed to determine how to quantify the preoperative peroneus longus graft diameter in ACL reconstruction.

## 2 Materials and methods

### 2.1 Patients

This research was a cohort retrospective study with consecutive sampling of ACL reconstruction patients in our center. The patients were diagnosed with total ACL rupture from February 2016 until October 2017 and completed an informed consent form to be included in this study. Thirty-nine patients underwent ACL reconstruction using peroneus longus tendon. Patient characteristics were recorded including body weight, height, Body Mass Index (BMI), and gender. It was reviewed and approved by the Medical and Health Research Ethics Committee with the ID number KE/FK/1314/EC/2017. This study has been registered in a publicly accessible database and having a unique identifying number: researchregistry5030.

The inclusion criteria was patient with isolated rupture of ACL at ages 16–45 years oldthat diagnosed using clinical examination (Lachmann test and anterior drawer test) and confirmed with knee Magnetic Resonance Imaging (MRI). ACL reconstruction was performed on patients with grade 3 and 4 ACL rupture (anterior drawer examination ≥11 mm side-to-side difference in anterior displacement), who still complained of pain and instability after conservative treatment for at least 3 months. Exclusion criteria was associated ligament injury, chondral damage, meniscus injury, fracture around the knee, and presence of pathologic condition in the lower extremity or an abnormal contralateral knee joint. This research work has been reported in line with the STROCSS criteria [9].

### 2.2 Arthroscopic technique

ACL reconstruction procedure was done by SR, a single senior knee surgeon. Patients was regionally anesthetized and laid in supine position. Tourniquet was applied to the thigh and inflated without leg elevation and exsanguination. Standard anterolateral and anteromedial portal were created. Surgeon confirmed the arthroscopy diagnostic for the ACL rupture. Peroneus longus autograft was harvested in ipsilateral leg.

Surgeon marked the skin incision location in 2–3 cm above and 1 cm behind the lateral malleolus. The skin was incised 3 cm longitudinal through the skin, subcutaneous tissue, and superficial fascia. The peroneus longus and peroneus brevis tendon were identified. Surgeon marked the location of tendon division which was 2–3 cm above the lateral malleolus. Distal part of the peroneus longus tendon to peroneus brevis tendon was sutured with end to side sutures. Peroneus longus tendon was stripped proximally until about 4–5 cm from the fibular head to prevent peroneal nerve injury using a tendon stripper. We measured the intraoperative graft diameter using the published ACL reconstruction graft diameter measurement guides (ConMed®, USA), with increments of 0.5 mm. Surgeon prepared the site for implantation of peroneus longus tendon. Surgeon cleared the intercondylar notch from fibrous tissue to make a good visualization during preparation of the tunnel. Some remaining ACL fiber was preserved as a reference for placement of the tunnel.

### 2.3 Statistical analysis

Statistical data were analysed by an independent statistician, and were considered significant if *p* < 0.05. Statistical analysis was performed with the computer program SPSS, version 25.0 (IBM Corp., Chicago). We determined normalized data using Shapiro-Wilk test and analysed them using Mann-Whitney, and Pearson correlation. Pearson correlation was used to find correlations between intraoperative graft diameter and patient's physical characteristics.

## 3 Results

During the period of the study, 42 patients underwent single bundle ACL reconstruction. Three patients were excluded and 39 patients fulfilled the inclusion criteria. There was 28 males and 11 females who received the peroneus longus tendon autograft. Subjects mean age was 25.10 ± 9.16. Mean weight of subjects was 71.23 ± 14.17. Mean height subjects was 169.13 ± 8.81. Mean BMI of subjects was 20.96 ± 3.44. Intraoperative graft diameter of peroneus longus was 8.56 ± 0.82. Patients’ characteristics was shown in Table 1.

**Table 1:** Subjects’ characteristics.

Characteristics	Mean	SD	Min	Max	N
Age [year]	25.10	9.16	16.00	50.00	
Sex					
Male					28 (51.3)
Female					11 (28.2)
Graft diameter [MM]	8.56	0.82	7.00	10.00	
Height [CM]	169.13	8.81	150.00	187.00	
Weight [KG]	71.23	14.17	43.00	110.00	
Body mass index (BMI) [KG/M^2^]	20.96	3.44	14.33	28.89	

ABBREVIATIONS: SD: Standard Deviation; Min: Minimum; Max: Maximum; N: Number of Subjects.

Correlation between intraoperative peroneus longus diameter with patient's gender was statistically significant with *p* = 0.000 (*p* < 0.05) and coefficient correlation was −0.547. Correlation between intraoperative peroneus longus diameter with patient's body weight was statistically significant with *p* = 0.014 (*p* < 0.05) and coefficient correlation was 0.391. Correlation between intraoperative peroneus longus diameter with patient's body height was statistically significant with *p=*0.009 (*p* < 0.05) and coefficient correlation was 0.413. Also, correlation between intraoperative peroneus longus diameter with patient's BMI was statistically significant with *p=*0.041 (*p < *0.05) and coefficient correlation was 0.328. Pre-operative and intra-operative correlation was shown in Table 2.

**Table 2:** Pre-operative and intra-operative correlation.

Pre-operative measurement	Peroneus longus graft diameter	
	Coefficient correlation	Significance
Gender	−0.547	0.000
Age	−0.104	0.528
Wieght	0.391	0.014
Height	0.413	0.009
Body mass index (BMI)	0.328	0.041

## 4 Discussion

ACL reconstruction is the most common surgery to repair ruptured ACL. There are many graft choices in ACL reconstruction. Graft decision is the most important preoperative consideration in ACL reconstruction. It can minimalize re-rupture incident and provide optimal knee stability after surgery.

Our study hypothesis was that patients' physical characteristics can influence the graft size in ACL reconstruction. We found significant correlation between peroneus longus graft diameter with patient's gender, height, body weight, and BMI. A previous study showed that height is the most influential measurement for the graft size diameter [10]. Theme et al. reported a correlation between patient's BMI and hamstring graft size diameter [11].

Xiaoxiao et al., in 2018 explained that weight, height, and duration of injury were variables which could determine the diameter of peroneus longus graft and could be used as important information before ACL reconstruction [12].

Hormone Replacement Therapy (HRT) such as estrogen that was consumed by women in their menopause period was found to cause a decrease in tendon diameter compared to women in their menopause period who did not consume HRT [13]. A study in Caucasian patients found that the length and diameter of the hamstring tendon could be predicted by anthropometric assessment. The diameter could be predicted by the patient's height which the smaller diameter was found more in female patients [14]. Based on research conducted in Iraq involving 178 patients, result showed that body weight, height, BMI, and length of the graft would affect the diameter of the graft [15].

Tendons are containing proteoglycans which are associated with collagen fibrils. The proteoglycans deterioration is accompanied by increasing in fibril diameter and age. The smaller diameter of the fibrils, the tendons are more flexible and resistant. This is due to larger surface area which produces greater interactions between fibrils. However, the larger diameter of the fibrils, the tendons are stronger. This is due to an increasing in intra-molecular crosslink density. The diameter of the fibrils are increase as the body is developing and decrease as the body is aging. The fibrils also decrease in extremities that are rarely used. However, changes in the diameter of the fibrils did not coincide with changes in the diameter of the tendon [16]. This pattern is also supported by a study conducted by Kivi et al. that age did not affect the diameter of the hamstring tendon. Body weight, height, and BMI were the factors that were the diameter of the tendons, in which the larger diameter of the tendon was found prominently in male patients [15].

Based on research by Park et al. involving 296 Asian patients, the diameter of the hamstring tendon could be distinguished based on height, weight, BMI, and sex in both the athlete group and non-athlete group [17].

Research showed that increased Insulin-like Growth Factor 1 (IGF-1) could increase collagen synthesis [14]. Another study also added that Growth Hormone (GH) could increase IGF 1 systemically which an increasing local mRNA expressions of 1Ea IGF in tendon tissue [18]. The increase of local IGF 1 would stimulate the expression of collagen in the striated muscles and tendons which caused an increase in the strength of this collagen [16].

Pereira et al. found that body weight, age, and BMI had no effect on the diameter of the hamstring tendon. Meanwhile, height was the main predictor of tendon length and diameter [19].

Body weight was found to affect the diameter of the hamstring and peroneus longus. Song et al. stated that the graft diameter of the hamstring tendon could be predicted from body height, weight, and duration of injury with regression coefficient of 0.225. This indicated 22.5% of the variance in the diameter of the hamstring tendon graft could be predicted by these three predictors.

According to the study of Ho et al., determination of preoperative factors in the form of anthropometric weight data showed a strong and significant relationship with r = 0.24, *p* < 0.01 in the male group and r = 0.51, *p* < 0.01 in the female group [20]. Another study by Goyal et al. did not show consistent results and did not include the variable of body height. Body weight was significantly associated with the diameter of *gracilis* tendon grafts and quadrupled tendons but was not significantly associated with the diameter of the *semitendinosus* tendon graft [5].

A prospective study by Çeliktaş et al. also stated that body weight had low predictive value for determining graft diameter (R^2^ = 0.157) [21]. The study by Loo et al. and Boisvert et al. also showed that weight as a preoperative anthropometric factor was not recommended to be used as a predictor of graft diameter [22,23].

There was no studies that addressed the mechanism of body weight could affect the diameter of the tendons as well as its relationship to graft diameter after ACL reconstruction. Previous study by Lee et al. showed that patients being obese/overweight had significant relationship with the recovery period of hamstring and quadriceps muscles [24].

Anatomical appearance of the Achilles tendon in obese patients looked larger than the appearance of Achilles tendon in normal patients [25]. However, this increase in tendon diameter was also followed by the rapid process of symptomatic degeneration, pain and decreased tendon function. Based on histopathological findings, turbidity in fibrillary collagen and abnormalities in the process of fat deposit remodeling were found, in which those could cause tendolipomatosis that resulted in architectural damage to the affected area. The impact of obesity was that it could disrupt both the body's supporting tendons and other non-supporting tendons [18].

Research for predictors on the peroneus longus diameter has not been widely performed. However, based on one study which used peroneus longus diameter to look for preoperative predictors, results showed that the predictors were body weight, height, and duration of injury [12].

The limitations of this study is low sample size and absence of comparison tendon autograft. However, some of the potential bias is limited by using a single surgeon, same rehabilitation protocol, and also same surgical procedure in all patients.

## 5 Conclusion

This cohort retrospective study showed significant correlation between patients’ physical characteristics (gender, height, weight, and BMI) with the diameter of intraoperative graft in ACL reconstruction using peroneus longus tendon autograft.

## Ethical approval

The informed consent form was declared that patient data or samples will be used for educational or research purposes. Our institutional review board also provide an ethical approval with KE/FK/1314/EC/2017 as the protocol number.

## Sources of funding

The authors declare that this study had no funding resource.

## Author contribution

Sholahuddin Rhatomy and Henry Tanzil conceived the study. Sholahuddin Rhatomy, Krisna Yuarno Phatama, Jeffrey Adrianus performed surgery, collected and analysed data. Henry Tanzil, Riky Setyawan, Camilla Amanda drafted the manuscript, analysed data, and critically revised the manuscript for important intellectual content. Tedjo Rukmoyo and Bambang Kisworo analysed data, prepared and drafted the manuscript. Sholahuddin Rhatomy, Henry Tanzil, and Riky Setyawan reviewed and edited manuscript. Sholahuddin Rhatomy facilitated all project-related tasks.

## Registration of research studies

This study has been registered in a publicly accessible database and having a unique identifying number: researchregistry5030.

## Guarantor

Sholahuddin Rhatomy, M.D.

## Data availability

The data used to support the findings of this study are available from the corresponding author upon request.

## Provenance and peer review

Not commissioned, externally peer reviewed.

## Consent

Written informed consent was obtained from the all of the patients for publication of this case report and accompanying images. A copy of the written consent is available for review by the corresponding author of this journal on request.

## Declaration of competing interest

The authors declare that there is no conflict of interest regarding the publication of this paper.
